# Predictive value of the systemic immune-inflammation index for cancer-specific survival of osteosarcoma in children

**DOI:** 10.3389/fpubh.2022.879523

**Published:** 2022-07-27

**Authors:** Haiping Ouyang, Zhongliang Wang

**Affiliations:** Department of Orthopedics, Chongqing Key Laboratory of Pediatrics, Ministry of Education Key Laboratory of Child Development and Disorders, National Clinical Research Center for Child Health and Disorders, China International Science and Technology Cooperation base of Child development and Critical Disorders, Children's Hospital of Chongqing Medical University, Chongqing, China

**Keywords:** pediatric osteosarcoma, systemic immune-inflammation index, cancer-specific survival, prognosis, event-free survival

## Abstract

**Background:**

Osteosarcoma (OS) is the primary malignant bone tumor that most commonly affects children and adolescents. Recent years effective chemotherapy have improved the 5-year survival in osteosarcoma patients to up to 60%-70%. Still, there is a lack of novel therapeutic strategies to enhance further survival. Our study aimed to evaluate the clinical significance of pretreatment inflammatory-based parameters, including PLT, NLR, and SII, as prognostic indicators of survival in pediatric osteosarcoma patients.

**Methods:**

A total of 86 pediatric osteosarcoma patients between 2012 and 2021 in the Department of Orthopedics or tumor Surgery of Children's Hospital affiliated to Chongqing Medical University were retrospectively analyzed. The clinicopathological variables and systematic inflammatory biomarkers, including NLR, PLR and SII, was performed by the A Receiver operating characteristic (ROC) curve and Cox proportional risk regression model. According to the results of multivariate analysis, a prognostic nomogram was generated, and the concordance index (C-index) was calculated to predict the performance of the established nomogram. The survival curve was plotted by the Kaplan-Meier method.

**Results:**

Univariate analysis showed that TNM stage, tumor size, NLR value, PLR value, SII value, neutrophil count and platelet count were related to CSS (*p* < 0.05). According to multivariate analysis, only TNM stage (*p* = 0.006) and SII values (*p* = 0.015) were associated with poor prognosis.To further predict survival in pediatric osteosarcoma patients, multivariate Cox regression analysis was used to predict cancer-specific survival at 1, 3 and 5 years. And constructed a nomogram model to predict children's CSS. The C-index of the nomogram is 0.776 (95%CI, 0.776–0.910), indicating that the model has good accuracy.

**Conclusion:**

Preoperative SII and TNM staging are independent prognostic markers for pediatric osteosarcoma patients. SII may be used in conjunction with TNM staging for individualized treatment of pediatric osteosarcoma patients in future clinical work.

## Introduction

Osteosarcoma is the primary malignant bone tumor that most commonly affects children and adolescents (1). The incidence rates of Osteosarcoma for all races and both sexes are 4.0 for the range 0–14 years and 5.0 for the content 0–19 years per year per million persons (2). Osteosarcoma exhibits a propensity to occur in the metaphysis of long bones and most commonly occurs in the distal femur (43%), proximal tibia (23%), or humerus (10%) (3). The lung is the most common site of metastasis, with over 85% of metastatic disease occurring there, while the bone is the second most common site of distant metastasis (3). Osteosarcomas may progress rapidly with poor prognosis and high mortality. Recurrence and metastasis are the significant causes of death and poor prognosis in children with Osteosarcoma. Recent years effective chemotherapy have improved the 5-year survival in osteosarcoma patients to up to 60%-70%. Still, there is a lack of novel therapeutic strategies to enhance further survival (4). The traditional approaches such as tumor size, metastasis, histological subtype, and tumor stage have been considered inaccuracy and inadequacy as prognostic parameters in routine clinical practice (5). Therefore, it is crucial to find reliable prognostic factors for pediatric osteosarcoma patients. Tumor-promoting inflammation has been recognized as an enabling characteristic of cancer (6). The interplay between local immune response and systemic inflammation plays vital roles in cancer progression and patient survival (7). Therefore, inflammatory parameters are strong candidates for predicting tumor prognosis. Measuring neutrophils, lymphocytes, and platelets on a total blood count may help understand systemic inflammatory responses. However, individual inflammatory parameters are susceptible to other factors, so a combination of inflammatory indicators such as neutrophil to lymphocyte ratio (NLR) platelet to lymphocyte ratio (PLR) may theoretically be more reliable. Recently, neutrophils, lymphocytes, and platelets have been used in a joined tool, a systemic immune-inflammation index (SII), to obtain the prognostic information in patients with various malignant tumors, such as hepatocellular carcinoma, esophageal squamous cell carcinoma, gastric cancer, non-small-cell lung cancer, colorectal cancer, and epithelial ovarian cancer (8–13). However, the relationship between these inflammatory markers and childhood osteosarcoma is poorly understood. Therefore, our study aimed to evaluate the clinical significance of pretreatment inflammatory-based parameters, including PLT,NLR and SII, as prognostic indicators of survival in pediatric osteosarcoma patients.

## Patients and methods

### Data source and data extraction

We conducted a retrospective analysis on pediatric osteosarcoma patients who underwent radical surgery or limb salvage surgery in the Department of Orthopedics or tumor Surgery of Children's Hospital affiliated to Chongqing Medical University from May 2012 to September 2021. The Ethics Committee approved this study of Children's Hospital Affiliated to Chongqing Medical University. Written informed consent was obtained for the study from the parents of the patients.

Inclusion criteria were: 1) pathologically diagnosed as Osteosarcoma. 2) No previous anticancer treatment. 3) Have detailed medical data and laboratory results, And 4) available follow-up. Exclusion criteria were: 1) pre-existing blood disorders. 2) There are inflammatory diseases such as infection before treatment. 3) Incomplete medical records and laboratory results; Or 4) use non-steroidal anti-inflammatory drugs, as this may interfere with blood tests. Finally, we collected the medical data of 86 pediatric osteosarcoma patients in our hospital.

### Data collection

We collected relevant clinicopathological data, including gender, age, region, medical insurance, primary tumor site, left and right sides, TNM stage, operation, chemotherapy, radiotherapy, metastasis, and survival time. Routine laboratory data included preoperative blood samples in determining neutrophil, lymphocyte, and platelet levels and calculating NLR, PLR, and SII indices. NLR and PLR were defined as the total number of neutrophils or platelets divided by the total number of lymphocytes. SII was calculated by the formula SII = (P × N)/L, where P,N and L represented peripheral blood plate, neutrophil, and lymphocyte counts, respectively.

### Follow-up

All pediatric osteosarcoma patients require regular follow-up after surgery. According to the institution's practice, we follow up once every 3 months in the first three years, once every 6 months in the fourth to 15 years, and once a year after that. Contact the patient by outpatient examination or telephone. Physical examination, blood test, surgical site X-ray, chest CT are routine clinical examination items in our hospital. Follow-up was completed until death or November 2021. Overall survival was considered the interval from surgery to the date of tumor-related death or loss of follow-up or last contact.

The event-free survival (EFS) period was defined as the time from the start of study treatment to metastasis, recurrence, or death. Cancer-specific survival (CSS) is the interval between the initial diagnosis of Pediatric Osteosarcoma and the occurrence of Pediatric osteosarcoma-specific death.

### Statistical analysis

All analyses were performed using SPSS 26.0 and R Software 4.1.0. Optimal prognostic cut-off values for NLR, PLR, and SII were calculated using the A Receiver operating characteristic (ROC) curve corrected by the Jorden index. These values were used as thresholds to group all patients above or below the points. The survival curve was plotted by the Kaplan-Meier method. Cox proportional risk regression model was used for univariate and multivariate analysis. Only significant prognostic parameters from the univariate Cox balanced risk model were included in the multivariate analysis to determine independent prognostic factors in pediatric osteosarcoma patients. Based on independent risk factors, nomograms that predicted CSS of pediatric osteosarcoma patients were built.The concordance index (C-index) was calculated to predict the performance of the established nomogram. *P* < 0.05 was considered statistically significant.

## Result

### Baseline patient characteristics

The basic characteristics of 86 pediatric osteosarcoma patients in this study are summarized in Table 1. Twenty-four of the pediatric osteosarcoma patients had died, and 62 of the pediatric osteosarcoma patients were alive. Fifty-four (62.8%) were boys, and 32 (37.2%) were girls. Forty-four (51.2%) pediatric osteosarcoma patients were from urban areas, and 42 (48.8%) pediatric osteosarcoma patients were from rural areas. 42 (48.8%) had health insurance, and another 44 (51.2%) did not. Eighty patients (93.0%) developed osteosarcomas in the extremities and six (6.98%) in the trunk. Forty-six patients (53.5%) had primary lesions in the left limb, and 34 patients (39.5%) had primary lesions in the right limb. According to TNM staging of Osteosarcoma, 28 (32.6%) had stage 1, 24 (27.9%) had stage II, 21 (24.4%) had stage III, and 13 (15.1%) had stage IV. Eighty-two (95.3%) had limb salvage surgery, while another four (4.65%) had amputation surgery. 74 (86.0%) had received chemotherapy, and 12 (14.0%) had not received chemotherapy. Only 4 (4.65%) received radiotherapy, and the remaining 82 (95.3%) did not. The mean tumor size was 71.0 mm. Platelet count, neutrophil count, and lymphocyte count were 407, 5.14, and 3.56, respectively. The mean values of PLR, NLR, and SII were 128, 1.61, and 677, respectively. No metastasis occurred in 78 patients (90.7%), and metastasis occurred in 8 patients (9.30%). Up to the last follow-up time, the mean survival time of 86 pediatric osteosarcoma patients was 33.6 months, including 21.2 months for the deceased pediatric osteosarcoma patients and 38.4 months for the living patients.

**Table 1:** Clinicopathological characteristics of children with OS.

	**ALL** ***N*** = **86**	**Dead** ***N*** = **24**	**Alive** ***N*** = **62**	**p**
Age	10.7 (2.93)	9.58 (3.59)	11.1 (2.55)	0.068
Sex				0.831
Male	54 (62.8%)	16 (66.7%)	38 (61.3%)	
Female	32 (37.2%)	8 (33.3%)	24 (38.7%)	
Region				1.000
Urban	44 (51.2%)	12 (50.0%)	32 (51.6%)	
Rural	42 (48.8%)	12 (50.0%)	30 (48.4%)	
Medical.insurance				0.392
No	42 (48.8%)	14 (58.3%)	28 (45.2%)	
Yes	44 (51.2%)	10 (41.7%)	34 (54.8%)	
Primary.site				0.179
Limb	80 (93.0%)	24 (100%)	56 (90.3%)	
Axial	6 (6.98%)	0 (0.00%)	6 (9.68%)	
lateral				0.057
Left	46 (53.5%)	10 (41.7%)	36 (58.1%)	
Right	34 (39.5%)	14 (58.3%)	20 (32.3%)	
Not pairs	6 (6.98%)	0 (0.00%)	6 (9.68%)	
Stage				<0.001
I	28 (32.6%)	2 (8.33%)	26 (41.9%)	
II	24 (27.9%)	2 (8.33%)	22 (35.5%)	
III	21 (24.4%)	9 (37.5%)	12 (19.4%)	
IV	13 (15.1%)	11 (45.8%)	2 (3.23%)	
Surgery				0.310
Limb salvage	82 (95.3%)	22 (91.7%)	60 (96.8%)	
Amputation	4 (4.65%)	2 (8.33%)	2 (3.23%)	
Chemotherapy				0.496
No	12 (14.0%)	2 (8.33%)	10 (16.1%)	
Yes	74 (86.0%)	22 (91.7%)	52 (83.9%)	
Radiotherapy				0.573
No	82 (95.3%)	24 (100%)	58 (93.5%)	
Yes	4 (4.65%)	0 (0.00%)	4 (6.45%)	
Size	71.0 (37.4)	89.9 (28.6)	63.7 (38.0)	0.001
PLR	128 (73.6)	166 (86.8)	113 (62.6)	0.011
NLR	1.61 (0.72)	1.90 (0.73)	1.49 (0.69)	0.021
SII	677 (464)	913 (462)	586 (436)	0.005
Platelet	407 (157)	474 (146)	381 (154)	0.013
Neutrophile	5.14 (1.48)	5.66 (1.24)	4.94 (1.53)	0.026
Lymphocyte	3.56 (1.17)	3.35 (1.43)	3.65 (1.06)	0.367
Metastasis				1.000
No	78 (90.7%)	22 (91.7%)	56 (90.3%)	
Yes	8 (9.30%)	2 (8.33%)	6 (9.68%)	
Survival. months	33.6 (23.4)	21.2 (21.9)	38.4 (22.3)	0.002

### ROC curve analysis of inflammatory indices of pediatric osteosarcoma patients

**Figure 1:**
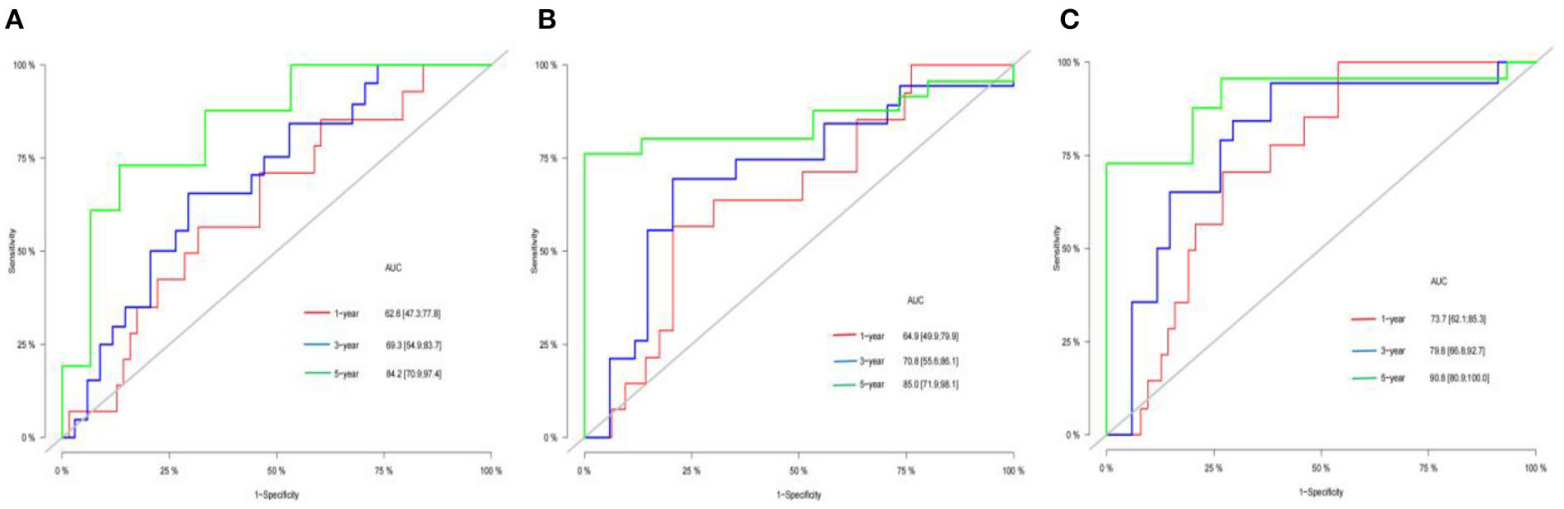
AUC of the NLR(**A)**, PLR**(B)**, and SII**(C)** for 1-, 3-, 5-year CSS of children with OS.

**Figure 2:**
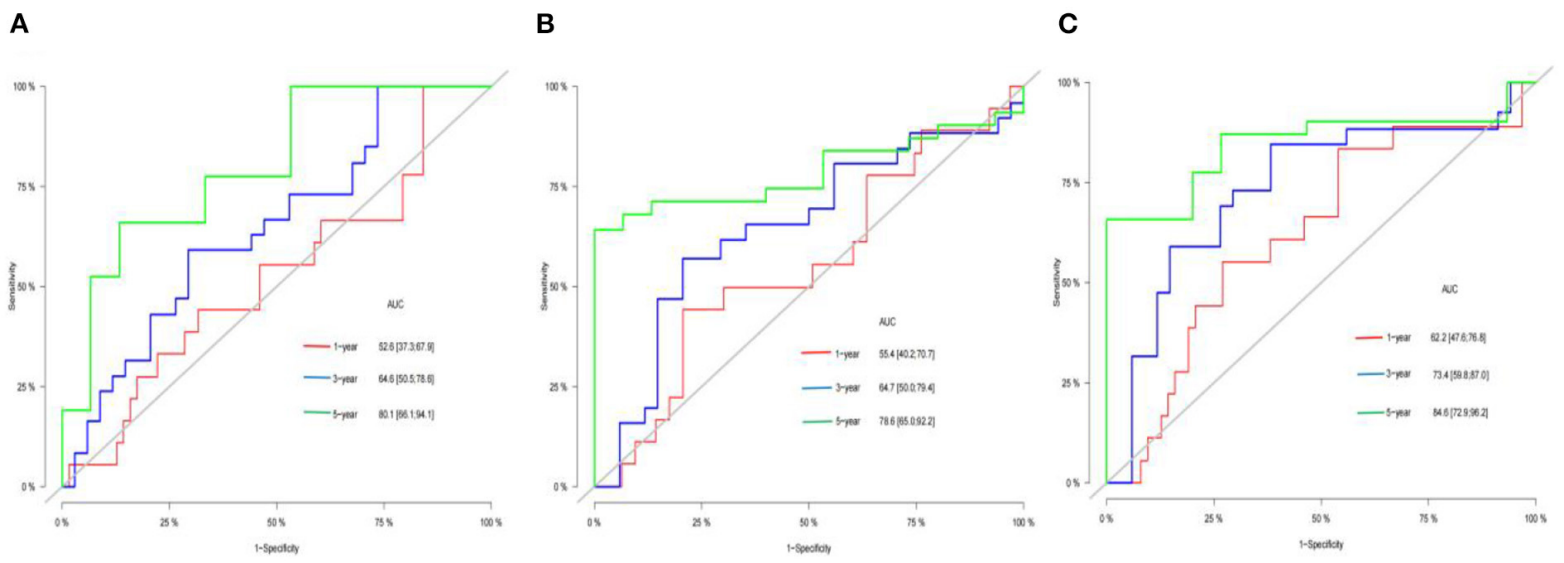
AUC of the NLR**(A)**, PLR**(B)**, and SII**(C)** for 1-, 3-, 5-year EFS of children with OS.

NLR, PLR, and SII were used to predict 1, 3 and 5-year event-free survival and cancer-specific survival in pediatric osteosarcoma patients. The accuracy of NLR, PLR and SII in predicting cancer-specific survival and event-free survival at 1, 3 and 5 years were shown in Figures 1, 2, respectively, by ROC curve analysis. With the extension of time, AUC gradually increased in Table 2. The optimal cut-off point was 0.80(NLR), 97.9(PLR)and 565(SII) according to ROC analysis. Among 86 included patients, NLR≥0.80, PLR≥97.9, SII≥565 were considered as high groups based on the above cut-off results.

**Table 2:** AUC of the NLR, PLR, and SII for 1-, 3-, 5-year CSS and EFS of children with OS.

	**AUC**	**95%CI**
NLR 1 year CSS	0.626	0.473–0.778
NLR 3-year CSS	0.693	0.549–0.837
NLR 5-year CSS	0.842	0.709–0.974
PLR 1 year CSS	0.649	0.449–0.799
PLR 3-year CSS	0.708	0.556–0.861
PLR 5-year CSS	0.850	0.719–0.981
SII 1 year CSS	0.731	0.621–0.853
SII 3-year CSS	0.798	0.668–0.927
SII 5-year CSS	0.908	0.809–1.000
NLR 1 year EFS	0.526	0.373–0.679
NLR 3-year EFS	0.646	0.505–0.786
NLR 5-year EFS	0.801	0.661–0.941
PLR 1 year EFS	0.554	0.402–0.707
PLR 3-year EFS	0.647	0.500–0.794
PLR 5-year EFS	0.786	0.650–0.922
SII 1 year EFS	0.622	0.476–0.768
SII 3-year EFS	0.734	0.598–0.870
SII 5-year EFS	0.846	0.729–0.962

### Cancer-specific survival and event-free survival

**Figure 3:**
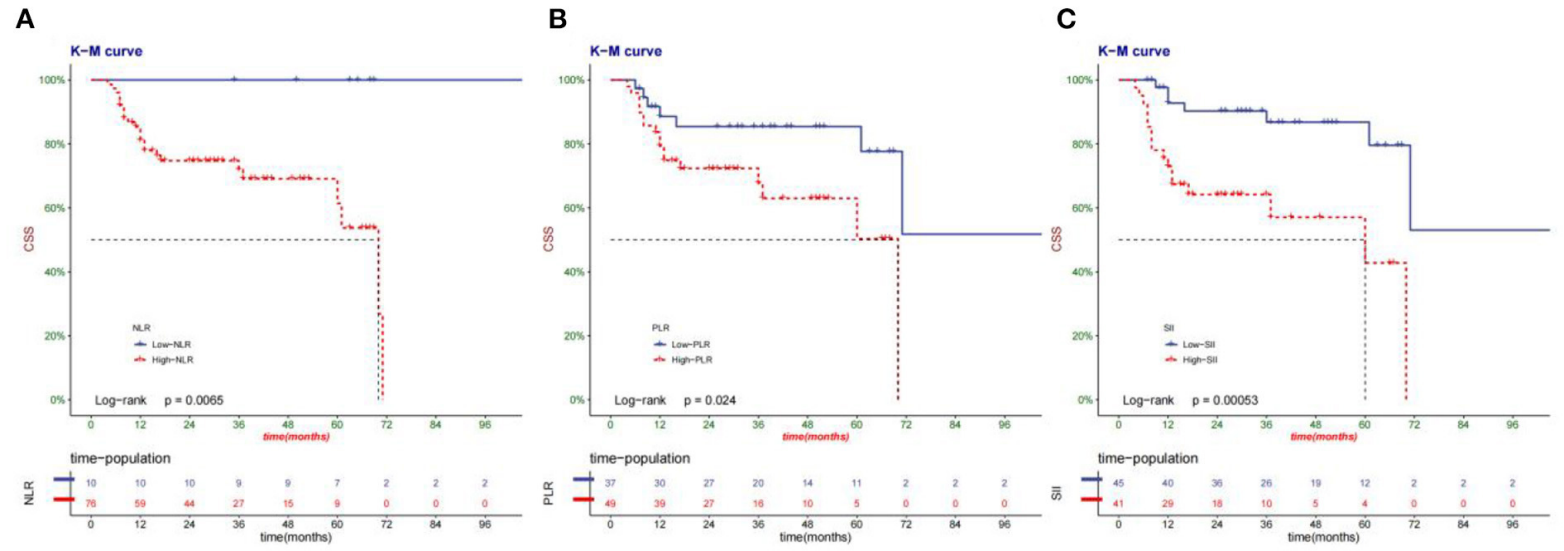
Kaplan-Meier curve of the CSS of patients according to NLR**(A)**, PLR**(B)**, and SII**(C)** group.

**Figure 4:**
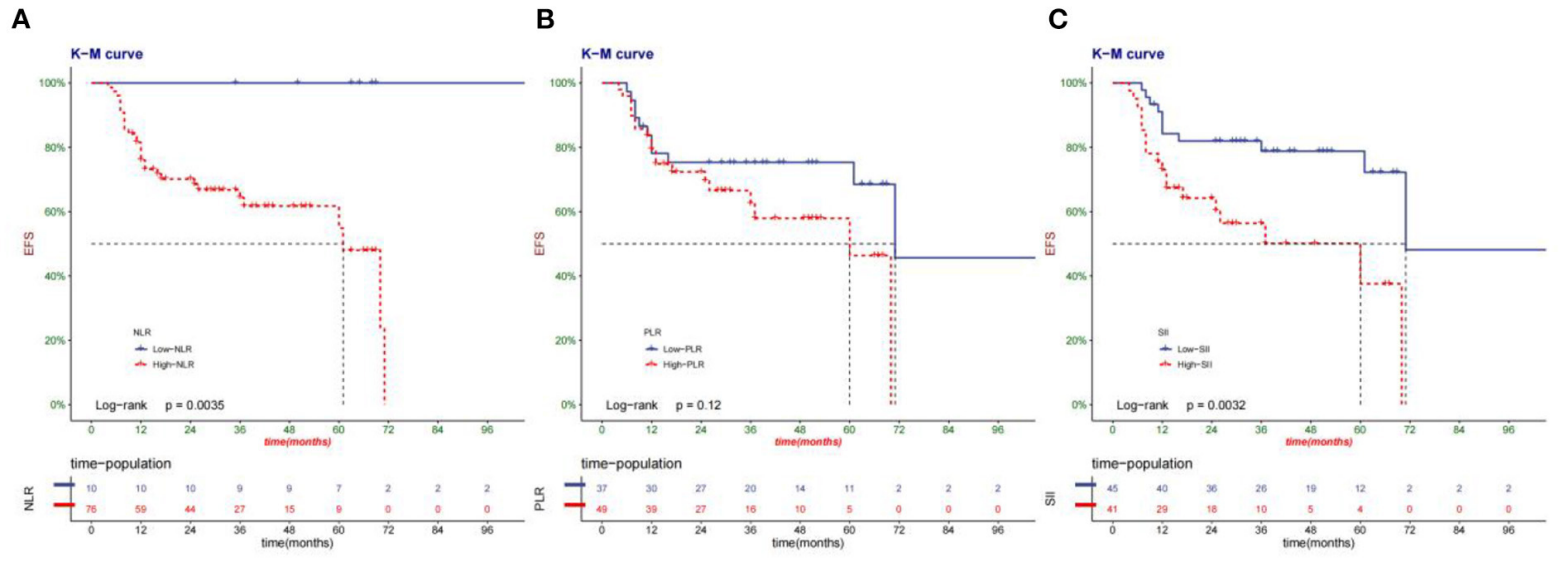
Kaplan-Meier curve of the EFS of patients according to NLR**(A)**, PLR**(B)**, and SII**(C)** group.

The median survival time of patients in this group was 33.6 months. Compared with high-NLR, PLR, and SII, low-NLR, PLR, and SII had a higher cancer-specific survival rate (Figure 3). Low-NLR, PLR, and SII have higher event-free survival than high-NLR, PLR, and SII (Figure 4).

### Univariate and multivariate cox regression analysis

**Figure 5:**
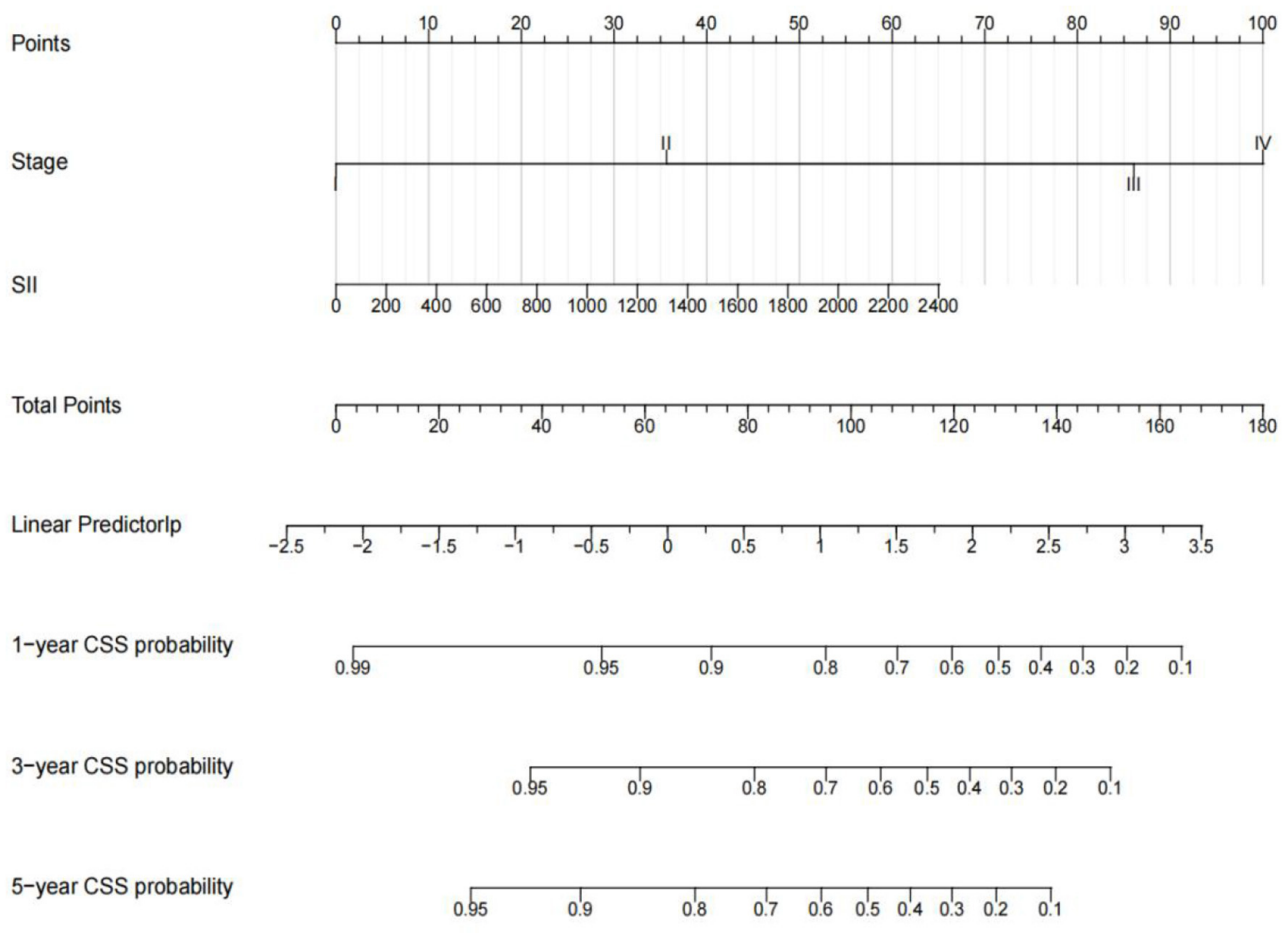
Nomograms for 1-, 3-, 5-year CSS of children with OS. Input individual patient variables. Each variable corresponds to a point, and the point of all variables can be added to find the corresponding total point. Below the total point is the survival rate for each patient.

Univariate analysis showed that TNM stage, tumor size, NLR value, PLR value, SII value, neutrophil count and platelet count were related to CSS (*p* < 0.05). In contrast, age, sex, region, health care, primary site, and laterality were not associated with CSS. According to multivariate analysis, only TNM stage (*p* = 0.006) and SII values (*p* = 0.015) were associated with poor prognosis, while NLR and PLR were not (Table 3). To further predict survival in pediatric osteosarcoma patients, multivariate Cox regression analysis was used to predict cancer-specific survival at 1, 3 and 5 years. And constructed a nomogram model to predict children's CSS (Figure 5). The C-index of the nomogram is 0.776 (95%CI, 0.776–0.910), indicating that the model has good accuracy.

**Table 3:** Univariate and multivariate analyses of CSS.

	**Univariate**			**Multivariate**		
	**HR**	**95%CI**	**P**	**HR**	**95%CI**	**P**
Age	0.89	0.78–1.02	0.097			
Sex						
Male	Reference					
Female	1.1	0.46–2.64	0.825			
Region						
Urban	Reference					
Rural	1.4	0.6–3.28	0.437			
Medical.insurance						
No	Reference					
Yes	0.59	0.26–1.34	0.212			
Primary.site						
Limb	Reference					
Axial	0	0–Inf	0.998			
lateral						
Left	Reference					
Right	1.71	0.74–3.98	0.211			
Not pairs	0	0–Inf	0.998			
Stage						
I	Reference			Reference		
II	3.48	0.31–38.79	0.31	3.322	0.298–37.08	0.329
III	17.68	2.23–140.52	0.007	18.106	2.273–144.207	0.006
IV	38.48	4.92–300.73	0.001	28.818	3.647–227.687	0.001
Surgery						
Limb salvage	Reference					
Amputation	1.96	0.45–8.44	0.369			
Chemotherapy						
No	Reference					
Yes	2.82	0.62–12.82	0.179			
Radiotherapy						
No	Reference					
Yes	0	0–Inf	0.997			
Size	1.02	1.01–1.03	0.003			
PLR	1.01	1–1.01	0.001			
NLR	2.07	1.27–3.39	0.004			
SII	1	1–1.001	0.001	1.001	1–1.002	0.015
Platelet	1	1–1.01	0.015			
Neutrophile	1.47	1.11–1.95	0.008			
Lymphocyte	0.73	0.51–1.06	0.102			

## Discussion

Osteosarcomas may progress rapidly with poor prognosis and high mortality. Recurrence and metastasis are the major causes of death and poor prognosis in children with Osteosarcoma. A new inflammatory indicator, the systemic immune inflammation index (SII), which combines inflammatory markers such as lymphocytes, neutrophils, and platelet count, has recently emerged and has been shown to predict poor prognosis in patients with hepatocellular carcinoma (8). This study evaluated preoperative systemic inflammatory markers in pediatric osteosarcoma patients, including SII PLR NLR, to understand the relationship between these markers and prognosis and survival in pediatric osteosarcoma patients. Univariate analysis showed that TNM stage, tumor size, NLR value, PLR value, SII value, neutrophil count and platelet count were related to CSS. Multifactorial analysis showed that only the TNM stage and SII values were associated with poor prognosis rather than NLR and PLR. Event-free survival and cancer-specific survival at 1, 3 and 5 years were higher in the low SII group than those in the high SII group.Based on the comprehensive indicators of peripheral blood neutrophil, platelet and lymphocyte count,the survival and prognosis value of SII for cancer patients may be derived from the function of these three cells, and there is increasing evidence that neutrophil and platelet's increase are related to carcinogenesis (14–17). Neutrophils not only promote the invasion of cancer cells Value-added and transfer, but also can help the cancer cells evade immune surveillance (18). Platelet protects cancer cells from immune clearance and promote their stranded in endothelial cells, to support the establishment of secondary lesions (19). In contrast, lymphocytes play an important role in the tumor defense by inducing cell death and inhibiting cell proliferation and migration (20). These mechanisms will help us better understand the role of neutrophil platelets and lymphocytes in cancer and their relationship with immunity and inflammation. Liu et al. concluded that elevated NLR PLR was associated with poor prognosis of Osteosarcoma, but they did not analyze the relationship between SII and prognosis of Osteosarcoma (21). Compared with PLR and SII, Yang et al. showed that NLR was a more reliable predictor of survival of Osteosarcoma, and no independent correlation was found between SII and survival of patients with Osteosarcoma (22). It should not be ignored that the difference in the efficacy of predictors in the literature may be due to cancer staging. Huang et al. suggested that high SII was an independent prognostic marker of postoperative survival of Osteosarcoma, which was consistent with our results (23). Another major difference between this study and the above studies (21, 23) is that the subjects are pediatric osteosarcoma patients, while the above studies are mainly adult osteosarcoma patients.This makes this study more significant in predicting the prognosis of pediatric osteosarcoma patients.The current treatment methods for Osteosarcoma are mainly chemotherapy, surgery and radiotherapy. Standard systemic therapy includes methotrexate based chemotherapy, including doxorubicin cisplatin and ifosfamide. Meta-analyses show that triple therapy is superior to double therapy and the importance of using high doses of methotrexate (24). Surgical resection after induction of chemotherapy is the standard for local control of osteosarcoma.Biopsies are performed at the time of diagnosis to confirm the pathological diagnosis and retrospective data suggest that local control is better when biopsies are performed by the same surgeon at a center experienced in surgical excision (25). With current treatment, about three-quarters of the patients diagnosed with Osteosarcoma are cured, and 90% to 95% of patients diagnosed with Osteosarcoma can be successfully treated by limb salvage surgery instead of amputation (26). Osteosarcoma is not a radiation sensitivity diseases. Therefore, radiotherapy is not considered a clear line of resectable tumors treatment. Instead, it is primarily used as a supplementary stage after marginal or incomplete resection, or for the final treatment of unresectable disease.In intratumoral or non-operative cases, patient who received adjuvant radiotherapy at the primary site had better overall survival than those who didi not receive radiotherapy (27).

However, in recent years, the 5- and even 10-year survival rates for pediatric osteosarcomas have not made breakthrough progress, so we need to find simple, easy, low cost and reliable non-invasive biochemical markers to predict the long-term prognosis of patients with Osteosarcoma in children. SII may give us a new direction to predict the survival rate of children patients with Osteosarcoma in different time. This may provide a new train of thought for clinicians to treat patients and further improve the long-term survival of patients. More studies are needed to determine the exact value of SII in pediatric osteosarcoma patients. However, the study has some limitations. First, we conducted a retrospective single-center study, and the sample size is relatively small. More studies are needed to confirm our results further. Second, although the predictive value of SII is confirmed, we did not compare the discriminative power of SII with other prognostic markers, such as PNI and CRP. Third, the patients are mainly from southwest China, which may lead to selection bias. More pediatric osteosarcoma patients from all over China are needed to study the relationship between SII and prognosis.

## Conclusion

This study is a retrospective study involving 86 pediatric osteosarcoma patients. Our results confirm that preoperative SII and TNM staging are independent prognostic markers for pediatric osteosarcoma patients. SII may be used in conjunction with TNM staging for individualized treatment of pediatric osteosarcoma patients in future clinical work. However, multicenter prospective studies and more patients are needed to validate our results.

## Data availability statement

The original contributions presented in the study are included in the article/supplementary material, further inquiries can be directed to the corresponding author.

## Ethics statement

The studies involving human participants were reviewed and approved by the Ethics Review Board of the Children's Hospital of Chongqing Medical University. Written informed consent to participate in this study was provided by the participants' legal guardian/next of kin.

## Author contributions

HO and ZW designed the study, collected and analyzed the data, drafted the initial manuscript, revised the article critically, reviewed and edited the article. All authors approved the final manuscript.

## Conflict of interest

The authors declare that the research was conducted in the absence of any commercial or financial relationships that could be construed as a potential conflict of interest.

## Publisher's note

All claims expressed in this article are solely those of the authors and do not necessarily represent those of their affiliated organizations, or those of the publisher, the editors and the reviewers. Any product that may be evaluated in this article, or claim that may be made by its manufacturer, is not guaranteed or endorsed by the publisher.
